# ASSOCIATION OF KIDNEY MARKERS AND DUAL TRAJECTORIES OF COGNITIVE AND PHYSICAL FUNCTION

**DOI:** 10.1093/geroni/igae098.1451

**Published:** 2024-12-31

**Authors:** Aman Shrestha, Michelle Shardell, Chixiang Chen, Stephen L Seliger, Charles Ginsberg, Lindsay Miller, Teresa Tian, Eleanor Simonsick

**Affiliations:** University of Maryland School of Medicine, Baltimore, Maryland, United States; University of Maryland School of Medicine, Baltimore, Maryland, United States; University of Maryland School of Medicine, Baltimore, Maryland, United States; University of Maryland School of Medicine, Baltimore, Maryland, United States; University of California San Diego, La Jolla, California, United States; University of California San Diego, La Jolla, California, United States; National Institute on Aging, Baltimore, Maryland, United States; National Institute on Aging, Baltimore City 0400 Baltimore City, Maryland, United States

## Abstract

The interplay between kidney health and cognitive-physical function has garnered increased attention. Dual trajectories consider simultaneous longitudinal changes in cognitive-physical function. Among participants in the Health, Aging and Body Composition Study with initially fast gait and high cognitive function, we assessed associations between baseline kidney function markers and joint longitudinal cognition-gait trajectories. Grouped-based trajectory analysis performed on modified mini-mental state (years 3, 5, 8, 10) and 20-meter usual gait speed (years 3–6, 8, 10) yielded three dual trajectory groups: Group 1 (n=660; exhibited sustained superior longitudinal cognitive-physical performance), Group 2 (n=744; high sustained cognition and initially lower, steadily declining gait), and Group 3 (n=498; lowest initial cognitive-physical performance, both steeply declining over time). Four sequential multinomial regression models were built. Model 1 showed a higher estimated glomerular filtration rate (ml/min/1.73m2) to be associated with lower odds of Group 3 versus Group 1 (odds ratio[OR]=0.98, 95% confidence interval [CI]: 0.97–0.99) after adjusting for covariates. Model 2 estimated 19% greater odds of Group 2 per log-transformed urine albumin-to-creatinine ratio (mg/g) (OR=1.19, 95%CI: 1.05–1.35) and 35% greater odds of Group 3 (OR=1.35, 95%CI: 1.17–1.55) versus Group 1. In Model 3, 25-hydroxyvitamin D was not significantly associated with cognitive-physical trajectory. Model 4 added parathyroid hormone, alpha-klotho, and fibroblast growth factor 23 (FGF23) (p=0.044 Model 4 versus 3); FGF23 was significantly associated with cognitive-physical trajectory (Group 3 versus 1 per log-transformed FGF23 [pg/mL]: OR=1.51, 95%CI:1.00–2.28). Findings support a potential role of kidney health in dual cognitive-physical performance among initially healthy older adults.

